# A model of DNA damage response activation at stalled replication forks by SPRTN

**DOI:** 10.1038/s41467-019-13610-7

**Published:** 2019-12-12

**Authors:** Christopher Bruhn, Marco Foiani

**Affiliations:** 1 IFOM (Istituto FIRC di Oncologia Molecolare), Via Adamello 16, 20139 Milan, Italy; 2 Università degli Studi di Milano, 20122 Milan, Italy

**Keywords:** DNA damage and repair, DNA replication

## Abstract

The process of DNA replication is threatened by many factors, including DNA lesions, and machineries acting as obstacles. Here we discuss and speculate on a recently proposed mechanism of DNA damage response activation in response to lesions that challenge the progression of DNA replication forks.

DNA lesions are particularly damaging during DNA replication as they interfere with replication fork progression and can cause replication fork stalling and collapse^1^. Replication fork stalling triggers the ATR-CHK1 pathway, a specialized DNA damage response (DDR) branch controlling the integrity of replicating chromosomes. This safeguard process acts locally, by stabilizing stalled replication forks to resolve topological constraints, and globally by influencing the firing of replication origins, cell cycle progression and dNTP synthesis.

ATR-CHK1 activation relies on the accumulation of single-stranded DNA (ssDNA), which can be generated by prolonged fork stalling^2^. CHK1 travels with forks and is therefore a directly available ATR target^3^. ATR-dependent CHK1 phosphorylation results in its activation by displacement of the inhibitory C-terminal domain and its release from chromatin to phosphorylate targets such as CDC25A^4^. Some endogenous events that interfere with replication fork progression do not produce long stretches of ssDNA, suggesting alternative modes of ATR-CHK1 activation^5^.

## SPRTN resolves DNA–protein crosslinks (DPCs)

A common block encountered by replication forks are DPCs. DPCs can originate from covalent stabilization of enzyme–DNA intermediates (topoisomerases) and chemical crosslinking of DNA-binding proteins (formaldehyde adducts)^6^. The removal of these lesions involves (1) crosslink reversal by specialized enzymes (TDP1, TDP2), (2) non-specific protease degradation by the protein component (SPRTN) and (3) nuclease-mediated removal of the DNA component (MRN complex). These mechanisms act together with classical DNA repair pathways. DPCs may not produce RPA-coated ssDNA filaments, particularly when hindering leading-strand synthesis^7^; however, they can induce fork stalling and topological stress also by generating unprogrammed termination sites where forks from adjacent replicons converge.

That SPRTN might be acting as a DPC-degrading protease was suggested by observations made in budding yeast with the SPRTN homolog Wss1^8^. Wss1 acts as a general protease towards chromatin-bound proteins, exhibiting two modes of activation: dsDNA activates its auto-cleavage activity, while ssDNA stimulates the activity of Wss1 towards other proteins. These separate activation modes likely prevent Wss1 from degrading proteins in a normal chromatin context. Homologs of Wss1 exist across metazoans, and SPRTN is a structural and functional human homolog of Wss1^9–12^. *Sprtn* is essential for mouse development, and hypomorphic *SPRTN* mutations underlying Ruijs–Aalfs syndrome cause hypersensitivity towards DPC-inducing agents, genome instability, hepatocellular carcinomas and progeroid symptoms^13,14^. Both SPRTN and Wss1 exhibit similar activation modes^10^. Upon DPC induction, de-ubiquitinated SPRTN is recruited to chromatin, suggesting the existence of a de-ubiquitination switch upstream of the DNA-dependent activation^10^. Consistent with a role in replication-associated DPC removal, SPRTN deficiency slows down replication fork progression in human cells exposed to formaldehyde^11,12^. SPRTN has also been demonstrated to localize to replication forks^11,14^, suggesting an immediate role in DPC recognition. The mode of SPRTN activation during replication-coupled DPC removal has been mechanistically characterized in vitro, implying that DPC-stalled replication forks may directly promote SPRTN activation^15^.

## DDR activation at stalled replication forks by SPRTN

A recent paper by Halder et al.^16^ proposes a model of DDR activation at stalled replication forks by SPRTN. The study shows that low SPRTN activity partially mimics replication phenotypes of CHK1 deficiency in terms of slow replication fork progression and excessive replication origin firing, suggesting a role for SPRTN during physiological CHK1 activation. SPRTN is shown to cleave the inhibitory C-terminus of CHK1 in vivo and in vitro, resulting in highly active N-terminal CHK1 fragments, which may contribute to DDR signaling. SPRTN activity is shown to promote the release of CHK1 from replication forks, and CHK1 was also found to enhance the activity of SPRTN through phosphorylation. These results offer several novel concepts discussed below.

## A potential ATR-independent shortcut for CHK1 activation by SPRTN

**Fig. 1:**
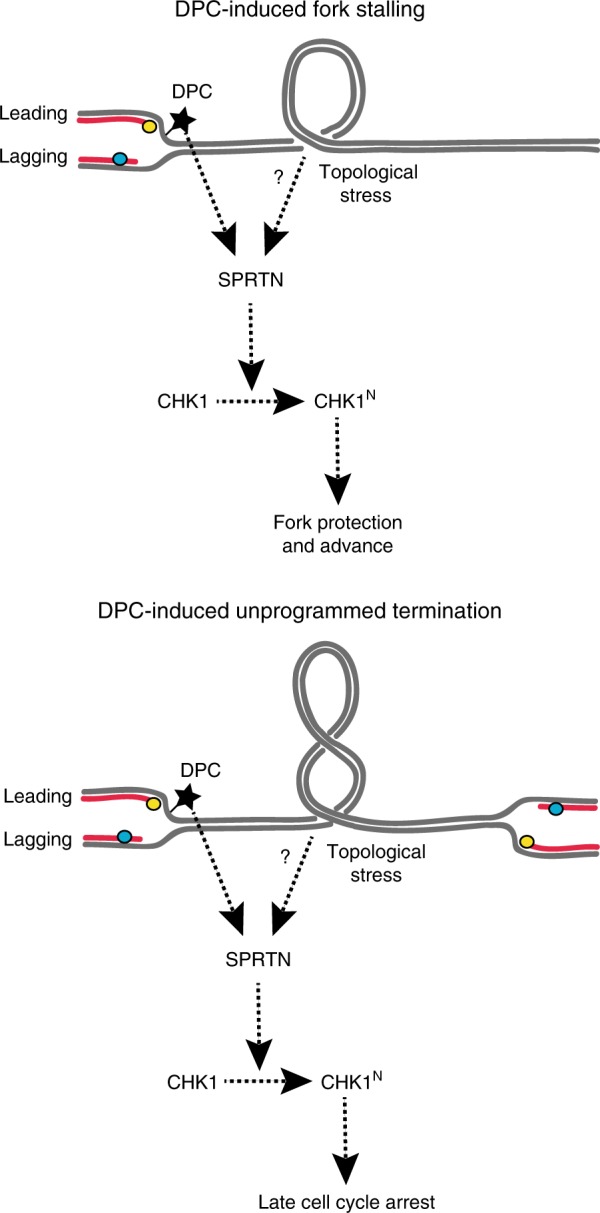
A model for DPC-induced Chk1 activation during replication. DPCs can induce fork stalling and topological stress, leading to activation of the SPRTN-CHK1 fork protection regulatory loop. Occasionally, DPCs can induce un-programmed termination sites where adjacent replicons converge without fusing, thus generating topological stress and preventing the completion of replication. Under these circumstances the SPRTN-CHK1 axis inhibits cell cycle progression to avoid segregation of partially replicated chromosomes.

Previous work has shown that when replisomes run into DPCs during leading-strand synthesis, DPC degradation is rapidly initiated, implying a function of the replisome in DPC sensing^17^. In their recent paper, Halder et al. suggest a role for SPRTN acting as a DNA damage sensor, and transmitting the damage signal by cleavage of CHK1^16^. It is possible that small stretches of ssDNA form due to transient uncoupling between leading- and lagging-strand synthesis, which could stimulate SPRTN activity. It is tempting to speculate that DPCs induce topological stress and perhaps accumulation of topoisomerase-chromatin adducts that provide a substrate for SPRTN activation, independent from ssDNA (Fig. 1). However, whether SPRTN can be regarded as a molecular sensor per se is still unclear, because SPRTN activation by DPCs requires its ubiquitin binding domain^15^, implying the existence of a signaling step between fork stalling and protease activation.

Does the activation of CHK1 by SPRTN represent an ATR-independent shortcut of CHK1 activation? SPRTN is activated at formaldehyde concentrations that do not induce classical CHK1 phosphorylation^10^, supporting the idea that SPRTN could act independently of the sensing mechanisms that culminate in ATR activation. The slow replication fork speed observed in SPRTN-deficient cells^12^ is not accompanied by exposure of long ssDNA stretches or CHK1 phosphorylation^16^, suggesting that DPCs could activate the DDR in an ATR-independent manner. Epistasis analysis placed SPRTN and CHK1 in the same pathway of replication fork progression^16^. However, complete CHK1 cleavage by SPRTN depends on the presence of ATR-dependent phosphorylation sites on CHK1, arguing that the SPRTN-CHK1 mechanism amplifies CHK1 activity downstream of ATR. Such dependency may underlie the principle of independent signal integration that exists to prevent inappropriate DDR activation. SPRTN-dependent CHK1 activation could therefore act as a weak CHK1 activator upon fork blockage on the leading strand, and become a signal amplifier following ATR recruitment during replication fork remodeling^18^ by converting phosphorylated CHK1 into a constitutively active form. Since cleavage removes the inhibitory C terminus of CHK1, N-terminal CHK1 fragments are likely resistant to phosphatase inactivation, which could significantly slow down checkpoint recovery.

## SPRTN as a mediator of basal replication-associated DDR activity

Halder et al. suggest that the SPRTN-CHK1 pathway mediates the basal DDR activity in S phase, which is an essential DDR function. SPRTN-dependent DPC cleavage was shown to facilitate fork advance together with translesion synthesis in DNA polymerases^15,17^, supporting the idea that SPRTN-CHK1 activation is a frequent event in S phase. The extended duration of replication fork stalling during the repair^15^ also argues for significant CHK1 activation and a local role of CHK1 in fork stabilization. Since DPCs might impede the fusion of convergent replicons at atypical replication termination sites, SPRTN-CHK1 may also induce a late cell cycle arrest^13^ to prevent segregation of partially replicated chromosomes. While the excessive replication origin firing in SPRTN mutant cells may also be explained by the inability to activate CHK1, it is also possible that the extensive fork pausing caused by SPRTN defects could elicit firing of origins that are passively replicated^19^. Sprtn-deficient mouse embryonic fibroblasts show no increase in spontaneous replication fork blockage^14^, suggesting that the contribution of SPRTN to DDR activation in S phase likely varies between cell types or species. In addition to CHK1, ATR activity is essential for genome stability in S phase and survival^20^, even in the presence of SPRTN. Thus, SPRTN-CHK1 likely contributes to DDR activity in S phase, but the relative contributions of physiological DPCs and SPRTN vs ATR to CHK1 activation need to be further investigated.

## Stimulation of SPRTN by CHK1 may establish a signal amplification loop

Halder et al. also suggest that mutual activation of SPRTN and CHK1 represents a signal amplification loop. Where SPRTN phosphorylation by CHK1 occurs is not known, but the transient enrichment of both CHK1 and SPRTN at the stalled replication fork would argue for a localized mechanism, creating a positive feedback loop of CHK1-SPRTN activation. The previously described stringency of SPRTN regulation by ubiquitination and DNA binding^10^ suggests that phosphorylation represents another regulation layer that restricts SPRTN activity to stalled replication forks rather than a global activation. Transiently maintaining high SPRTN activity after DPC removal may also help clear unwanted proteins on ssDNA (e.g. histones) during replication fork restart.

In conclusion, SPRTN represents a potential shortcut between DPC and DDR activation that may contribute to genome stability.
